# The potential role of serum expression profile of long non coding RNAs, Cox2 and HOTAIR as novel diagnostic biomarkers in systemic lupus erythematosus

**DOI:** 10.1371/journal.pone.0268176

**Published:** 2022-08-16

**Authors:** Rania H. Mahmoud, Nermeen A. Fouad, Enas M. Hefzy, Olfat G. Shaker, Tarek I. Ahmed, Hoda A. Hussein, Maha H. Nasr, Othman M. Zaki, Noha K. Abdelghaffar, Omayma O. Abdelaleem

**Affiliations:** 1 Department of Medical Biochemistry and Molecular Biology, Faculty of Medicine, Fayoum University, Fayoum, Egypt; 2 Department of Rheumatology and Rehabilitation, Faculty of Medicine, Fayoum University, Fayoum, Egypt; 3 Department of Medical Microbiology and Immunology, Faculty of Medicine, Fayoum University, Cairo, Egypt; 4 Department of Medical Biochemistry and Molecular Biology, Faculty of Medicine, Cairo University, Cairo, Egypt; 5 Department of Internal Medicine, Faculty of Medicine, Fayoum University, Fayoum, Egypt; 6 Department of Clinical Pathology, Faculty of Medicine, Damietta University, Damietta, Egypt; 7 Department of Clinical Pathology, Faculty of Medicine, Fayoum University, Fayoum, Egypt; Berhampur University, INDIA

## Abstract

**Background:**

The role of the long non-coding RNAs (lncRNAs) in the pathogenesis of systemic lupus erythematosus (SLE) is mostly unknown, despite increasing evidence that lncRNAs extensively participate in physiological and pathological conditions.

**Aim:**

To detect the level of lncRNA-Cox2, HOTAIR, IL-6, and MMP-9 in the serum of SLE patients and to correlate these levels with disease activity and patients’ clinical and laboratory data to evaluate the value of these biomarkers for SLE diagnosis and assessment of disease activity.

**Methods:**

Blood samples from 58 SLE patients, and 60 healthy controls (HCs) were used for detection of lncRNAs-Cox2 and HOTAIR expression levels by real-time polymerase chain reaction. Both IL-6 and MMP-9 serum levels were assayed by enzyme-linked immunosorbent assay. Lupus activity was assessed with the Systemic Lupus Erythematosus Disease Activity Index (SLEDAI).

**Results:**

The serum expression levels of lncRNA-Cox2 and HOTAIR were significantly up-regulated in SLE patients vs HCs (fold change [median (IQR) was 1.29(0.81–1.71, *P*<0.0001) and 2.68(0.95–3.67), *P* = 0.038) for lncRNA-Cox2 and HOTAIR, respectively. Serum levels of both IL-6 and MMP-9 were significantly high in SLE patients compared with HCs (*P≤*0.001 for each). The up-regulated lncRNA-Cox2 was positively associated with the presence of neurological manifestations in SLE patients (*P* = 0.007). Furthermore, HOTAIR expression level had significantly positive correlation with IL-6 (*r =* 0.578, *P*<0.0001), MMP-9 level *(r* = 0.762, *P*<0.0001), nephritis grades (*r =* 0.296, *P =* 0.024) and proteinuria (*r =* 0.287, *P =* 0.035). LncRNA-Cox2 showed sensitivity and specificity 72.4%, and 100.0% respectively. HOTAIR sensitivity was 60.3%, and specificity was 100.0%. By multiple logistic regression analysis, lncRNA-Cox2 and HOTAIR were found as SLE independent predictors.

**Conclusion:**

LncRNA-COX2 and HOTAIR can be used as new non-invasive biomarkers for the diagnosis of SLE.

## Introduction

Systemic lupus erythematosus (SLE) is a systemic poorly understood autoimmune disease that involves both the innate and acquired immune systems and manifested by the presence of immune complex deposition which lead to multiple organ damage [1]. SLE loses its immune tolerance to nuclear autoantigens and produces anti-nuclear antibodies (ANAs). The production of autoantibodies targets double-stranded DNA (dsDNA) and other nuclear autoantigens are essential characteristics of the disease [2].

Anti-dsDNA antibodies are seen in about 60% to 70% of SLE patients and have more than 95% specificity, it can correlate with disease activity and with the development of lupus nephritis. in addition, low levels of complement explain complement consumption and also correlate with disease activity. Markers of inflammation like erythrocyte sedimentation rate (ESR) and C-reactive protein (CRP) can be elevated during disease activity [3].

Long non-coding RNAs (lncRNAs) are long RNA (200 or more nucleotides) that does not code for any protein. Few lncRNAs are vital regulators of the immune reaction [4] and play roles in various immune-mediated inflammatory diseases as SLE [5]. They are expressed abundantly in almost all immune cells and mostly associated with inflammatory diseases, tumorigenesis, and neurological disorders [6]. There is a necessity to discover new biomarkers for diagnosis and prognosis of SLE due to the varied presentation of SLE patients and their impulsive disease course. Using lncRNAs as biomarkers is still extensively rare [7].

LncRNA-Cox2 does not code for the COX-2 protein although it is present near to the gene of COX-2 [8]. LncRNA-Cox2 is a dynamically regulated gene, which is induced by TLR (Toll-like receptors) ligands leading to both promotion and repression of inflammatory gene expression [4]. It is an early-responsive lncRNA, stimulated by TNF-α via activation of the nuclear factor-κB (NF-κB, an essential regulator of inflammatory and immune response) signaling pathway [8]. It may be implicated in the pathogenesis of the autoimmune diseases as it regulates many important immune genes as IL-6, TNF-α, and STAT3 [4, 9].

HOTAIR (Homeobox transcript antisense intergenic RNA) is involved in the development, progression and bad prognosis of many cancers [10]. HOTAIR may affect the pathogenesis of RA [11]. However, little is known about its expression and effect on other immune-related diseases like SLE. LncRNA-Cox2 can regulate the induction of inflammatory cytokines such as interleukin-6 (IL-6). These cytokines are implicated in SLE pathogenesis [12].

Many SLE specific lncRNAs have been associated with clinical markers such as ESR, CRP, antinuclear antibodies (ANAs) and low levels of complement factors C3 and C4. In spite of identifying these lncRNAs, very few of them have been functionally investigated in SLE till now. These lncRNAs include GAS5, MALAT1, TUG1, NEAT1, UCA1, XIST and THRIL [13].

IL-6 is over-produced from monocytes and B-cells, and it is considered as a major inflammatory mediator that participates in multiple regulatory pathways of chronic autoimmune diseases such as SLE [14]. Matrix metalloproteinase-9 (MMP-9) is included in the dysregulation of the immune system and inflammation. It may have a role in lupus nephritis pathogenesis and it could be a marker of monitoring disease activity and renal damage [12]. MMP-9 secretion was detected to be suppressed markedly by down-regulation of HOTAIR [15].

The current study aimed to assess the serum expression level of lncRNA-Cox2, HOTAIR, and their targets IL-6 and MMP-9, respectively in the sera of patients with SLE and to correlate their levels with the disease activity and patients’ clinical and laboratory data to evaluate the value of these biomarkers for diagnosis of SLE and assessment of disease activity.

## Patients and methods

This study was done on 58 patients with SLE who fulfilled the Systemic Lupus International Collaborating Clinics classification criteria (SLICC) for SLE [16]. Another 60 healthy controls (HCs) matched with the patients for age and sex and without any history of autoimmune diseases, chronic diseases, smoking and obesity were included in the study. Patients were recruited from the inpatient and the outpatient clinics of Rheumatology and Internal Medicine departments, Fayoum University Hospitals, Egypt, in the period from March 2020 to September 2020. Full history taking with full clinical examinations were done for all patients. Clinical manifestations of SLE were assessed. Laboratory investigations were done and diagnosis of Lupus nephritis was confirmed by renal biopsy [17]. Disease activity was assessed by the SLE disease activity index (SLEDAI) [18], and SLEDAI score was as follow: SLE patients with SLEDAI = 0 were considered to be in remission, SLE patients with SLEDAI from 1 to 5 were considered to have mild disease activity, SLEDAI from 6 to 10 were considered to have moderate disease activity, SLEDAI from 11 to 19 were considered to have high disease activity, very high disease activity was for SLEDAI more than 20. Also, assessment of Systemic Lupus International Collaborating Clinics/American College of Rheumatology (SLICC/ACR) Damage Index was done [19].

The study protocol had complied with the guidelines of the Helsinki Declaration. The study was revised and approved by Ethical Committee at the Faculty of Medicine, Fayoum University. Informed written consent was obtained from all subjects after the explanation of the study. Patients were excluded from the study if they had acute severe infections, other autoimmune diseases, suspected drug abuse or malignancy.

### Blood sample processing and serum IL6 and MMP9 assays

Blood samples were withdrawn from all study subjects. Samples collected in serum separator tubes were left for 15 min to clot. Then it was centrifuged for 10 min at 4000×*g*. The separated serum samples were divided into portions and stored at −80°C until used. These sera were used in detecting serological markers and routine investigations for SLE, serum IL-6, and MMP-9 levels as well as RNA extraction. Serum IL-6 and MMP-9 levels were evaluated using human ELISA kit provided by Koma Biotech Inc., Seoul, Korea, according to the manufacturer’s instructions. All sera were assayed on one day to stay away from inter-assay variations.

### LncRNA-Cox2 and HOTAIR expression assays in serum [RNA extraction, reverse transcription and quantitative real-time PCR (qRT-PCR)]

For assessment of lncRNA-Cox2 and HOTAIR expression in serum, total RNA was extracted from sera of SLE patients as well HCs using miRNeasy mini kit (Qiagen, Valencia, CA, USA) for purification of serum total RNA, including lncRNAs. Then, the reverse transcription was done by the RT2 First Strand Kit (Qiagen, Maryland, USA) as per the manufacturers’ instructions. The resulted cDNA was used for real-time PCR quantification using RT2 SYBR Green ROX q PCR Master Mix, RT2 IncRNA qPCR Assay (Qiagen, Maryland, USA). GAPDH, as a housekeeping gene, was used as an internal control gene for the qPCR reactions [20]. The primers’ assay numbers were as follows; lncRNA-Cox2 (LPH29557A), HOTAIR (LPH07360A), and GAPDH (LPH31725A). The level of expression of lncRNA-Cox2, as well as HOTAIR in each sample, was normalized to that of the GAPDH.

LncRNA-Cox2 and HOTAIR expression levels, expressed as fold change (FC), were calculated by the 2 ^− ΔΔCt^ method. Control FC values were set as 1. FC value >1 indicates up-regulation, while that <1 indicates down-regulation [21].

### Statistical analysis

Data was encoded and analyzed using the Statistical Package for the Social Sciences (SPSS) version 24. Quantitative data were summarized using minimum and maximum, mean, median, standard deviation (SD), while frequency and percentage were used for categorical data. For comparing data, the non-parametric Mann-Whitney test was used for quantitative variables and the Chi-square (χ2) test was used for categorical data. If the expected frequency is < 5, the exact test was used instead. Spearman correlation coefficient was used for correlations between quantitative variables. Multiple logistic regression analysis was used to test and identify lncRNA-Cox2 and HOTAIR as SLE independent predictors.

The receiver operating characteristic (ROC) curve was constructed and area under curve (AUC) analysis was performed to detect the best cut-off value of the tested parameters for case detection. Statistical significance was considered when *P-*value ≤0.05. Sample size was calculated using (G power version 3.0.10). Minimal sample size of patients was 53; assuming a power level of 0.80, alpha level of 0.05 and medium effect size of 0.55 for (COX-2 Fold change) between the study groups. Finally, sample size increased by 10% to reach 58 in patient group.

## Results

### The demographic, clinical, and laboratory data of all participants

In the current study there was no significant difference between SLE patients and HCs group regarding age and sex. The SLE patients enrolled in the study had disease duration 8.66±3.21 years, with SLEDAI was between 0–20 with mean 5.75±3.12. There was a significant difference in levels of hemoglobin, platelets, and total leucocytic count (TLC) (*P*<0.0001 each) in SLE patients compared with the HCs. In addition, there was a significant increase regarding serum levels of IL-6, MMP-9, ESR. The demographic and clinical features of all participants are given in **Table 1**.

**Table 1:** Demographic, clinical and laboratory data of systemic lupus erythematosus (SLE) patients.

Variables (M±SD or N%)	SLE patients (n = 58)	Healthy control (n = 60)	P-value
**Age/y**	41.60±9.67	39.90±9.07	0.327
**Sex**			
**Females**	48 (82.8)	42 (70)	0.103
**Males**	10 (17.2)	18 (30)	
**IL-6 (pg/ml)**	10.89±2.57	3.81±0.78	<0.0001*
**MMP-9 (ng/ml)**	183.90±45.23	59.55±9.78	<0.0001*
**ESR (mm/1st h)**	37.50±23.32	6.3±1.7	<0.0001*
**Hb (g/dl)**	9.87±1.98	12.07±1.13	<0.0001*
**TLC (×10** ^**3**^ **/mm** ^**3**^ **)**	4.58±1.22	6.23±1.44	<0.0001*
**PLT (×10** ^**3**^ **/mm** ^**3**^ **)**	153.34 ±77.79	255.3±82.0	<0.0001*
**Disease duration**	8.66±3.21		
**Proteinuria (gm)**	1.51±0.51		
**SLEDAI**	5.75±3.12		
**In remission**	15 (25.9)		
**Mild**	18 (31.0)		
**Moderate**	16 (13.6)		
**High**	6 (10.3)		
**Very High**	3 (5.2)		
**SLICC/ACR**	1.55±1.54		
**Arthritis**	33 (56.9)		
**Cutaneous manifestations**	28 (48.3)		
**Oral ulcers**	28 (48.3)		
**Photosensitivity**	22 (37.9)		
**Nephritis**			
**No nephritis**	3 (5.2)		
**Class II**	9 (15.5)		
**Class III**	3 (5.2)		
**Class IV**	25 (43.1)		
**Class IV\V**	5 (8.6)		
**Class V**	13 (22.4)		
**Leukopenia (<3000/μl)**	24 (41.4)		
**Thrombocytopenia (<15x10** ^**4**^ **/μl)**	21 (36.2)		
**Neurological manifestations**	18 (31.0)		
**ANA**	58 (100)		
**Anti dsDNA**	34 (58.6)		
**aCL**	18 (31%)		
**LACs (IgG and IgM)**	9 (15.5%)		
**Consumed C3 (<80 mg/dl)**	28 (48.3%)		
**Consumed C4(<12 mg/dl)**	22 (37.9%)		
**CRP positivity (> 6mg/L)**	6 (10.3%)		

Mann-Whitney U test and Chi-squared (χ2) test were used.

* Significant at *p* value<0.05.

SLE: systemic lupus erythematosus, SLEDAI: Systemic Lupus Erythematosus Disease Activity Index, SLICC/ACR: Systemic Lupus International Collaborating Clinics/American College of Rheumatology Damage Index, IL-6: Interleukin-6, MMP-9: Matrix Metalloproteinase-9, ESR: Erythrocyte sedimentation rate, Hb: hemoglobin, TLC: total leucocytic count, PLT: platelet count, ANA: antinuclear antibody, anti-dsDNA: anti-double stranded DNA, aCL: anticardiolipin, LACs: Lupus anticoagulants, C3: complement 3, C4: complement 4, CRP: C-reactive protein.

### Serum expression levels of lncRNA-Cox2 and HOTAIR in the study subjects

**Fig 1:**
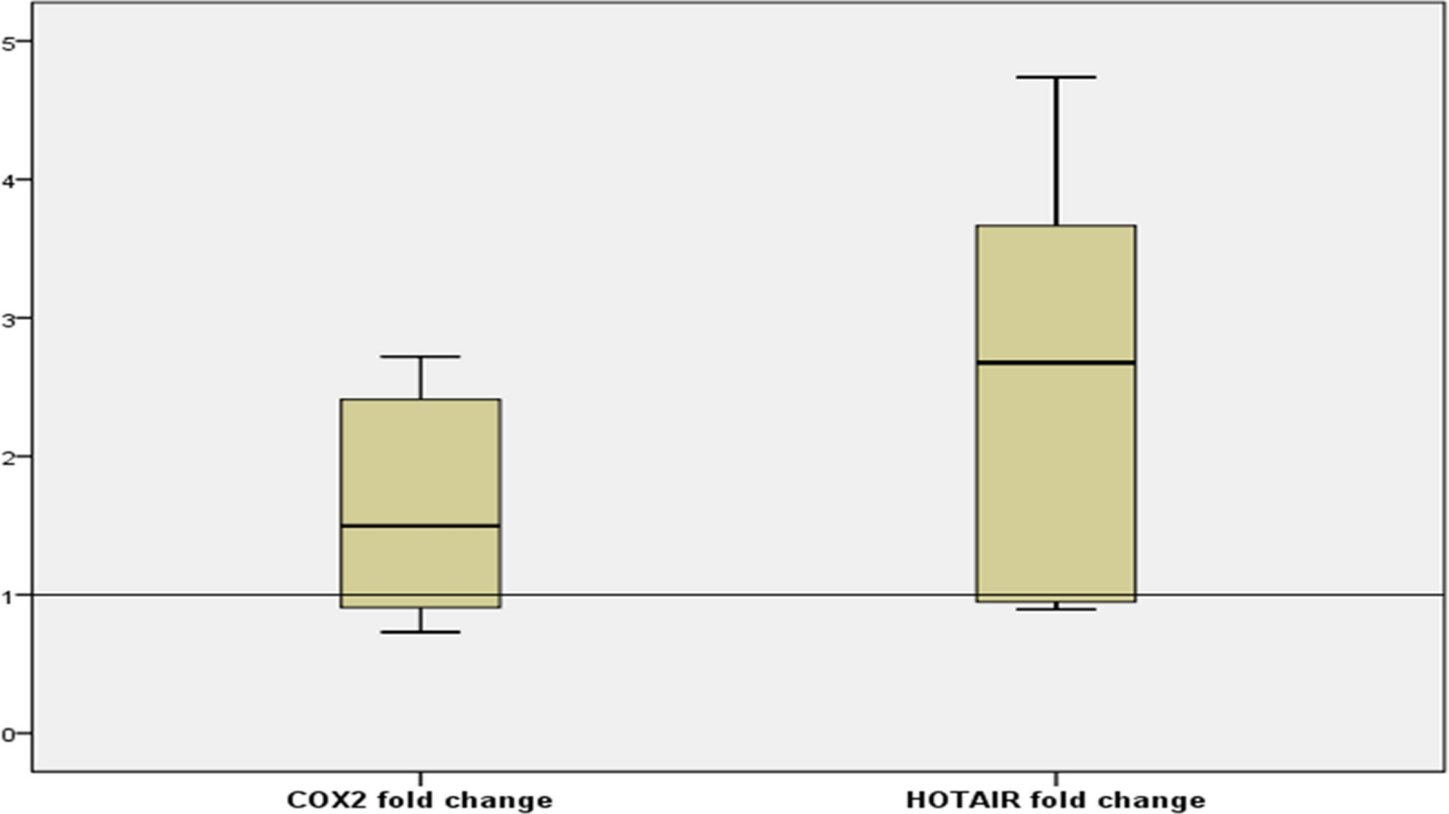
Comparison of expression of (a) serum lnc-Cox2 (b) serum HOTAIR (expressed as fold changes) between patients with SLE (58 patients) and healthy controls (60 subjects). The expression levels were analyzed by real-time quantitative PCR and normalized by GAPDH level. (a) Significantly increased expression of lnc-Cox2 in patients with SLE versus healthy controls (b) Significantly increased expression of HOTAIR in patients with SLE versus healthy controls.

Patients with SLE had significantly high expression levels of both lncRNA-Cox2 and HOTAIR in comparison to HCs. Expression levels were expressed as fold change. The fold change was [median (IQR); 1.29 (0.81–1.71, *P*<0.0001) and 2.68 (0.95–3.67), *P* = 0.038) for lncRNA-Cox2 and HOTAIR, respectively (**Fig 1**).

### Relation between serum lncRNA-Cox2 and HOTAIR levels and clinical manifestations and laboratory findings in SLE patients

A significantly increased expression of lncRNA-Cox2 was reported in SLE patients with neurological manifestations (P = 0.007). There was a marked increase in the serum expression level of HOTAIR in SLE patients with the presence of cutaneous manifestations, photosensitivity, and anti-cardiolipin antibodies IgG and IgM (aCL) (P <0.0001, 0.03, and 0.007) respectively (**Table 2**).

**Table 2:** Relation between serum lncRNA-Cox2 and HOTAIR levels and clinical manifestations and laboratory findings in systemic lupus erythematosus (SLE) patients.

	lncRNA-Cox2 Fold Change	HOTAIR Fold Change
**Arthritis**		
Yes (N = 33)	1.50 (1.21–2.18)	2.68 (0.95–3.64)
No (N = 25)	1.32 (0.90–2.41)	2.68 (0.95–3.67)
*P* value	0.660	0.844
**Cutaneous manifestations**		
Yes (N = 28)	1.51 (1.23–2.41)	3.64 (2.63–4.28)
No (N = 30)	1.31 (0.90–2.18)	0.96 (0.95–2.72)
*P* value	0.282	<0.0001*
**Oral ulcers**		
Yes (N = 28)	1.52 (1.07–2.43)	2.68 (0.95–3.65)
No (N = 30)	1.32 (0.91–2.18)	2.65 (0.94–3.67)
*P* value	0.358	0.744
**Photosensitivity**		
Yes (N = 22)	1.51 (0.90–2.43)	3.64 (0.95–4.33)
No (N = 36)	1.41 (0.91–2.18)	2.08 (0.95–2.95)
*P* value	0.682	0.03*
**Nephritis**		
No nephritis (N = 3)	1.62 (1.62–2.72)	3.67 (3.64–3.67)
Class II (N = 9)	1.50 (0.90–1.62)	2.72 (1.00–4.33)
Class III (N = 3)	1.23 (1.23–2.41)	3.64 (2.96–3.64)
Class IV (N = 25)	1.29 (0.91–2.18)	2.72 (0.95–3.67)
Class IV\V (N = 5)	0.81 (0.81–1.32)	0.92 (0.92–2.58)
Class V (N = 13)	1.71 (1.32–2.72)	2.58 (0.95–2.68)
*P* value	0.288	0.064
**Leucopenia (<3000/μl)**		
Yes (N = 24)	1.51 (1.21–2.18)	2.08 (0.95–3.31)
No (N = 34)	1.50 (0.90–2.43)	2.72 (0.95–3.67)
*P* value	0.716	0.439
**Thrombocytopenia (<15x10** ^**4**^ **/μl)**		
Yes (N = 21)	1.52 (1.23–2.18)	2.58 (0.96–3.67)
No (N = 37)	1.32 (0.91–2.41)	2.72 (0.95–3.64)
*P* value	0.466	0.764
**Neurological manifestations**		
Yes (N = 18)	1.07 (0.81–1.50)	1.76 (0.93–3.78)
No (N = 40)	1.67 (1.23–2.43)	2.72 (0.98–3.64)
*P* value	0.007*	0.153
**anti-dsDNA**		
Yes (N = 34)	1.50 (0.90–1.71)	2.72 (0.95–3.78)
No (N = 24)	1.85 (0.91–2.43)	1.00 (0.95–2.96)
*P* value	0.304	0.312
**aCL (IgG and IgM)**		
Yes (N = 18)	1.32 (0.91–1.71)	0.95 (0.95–2.58)
No (N = 40)	1.51 (1.06–2.41)	2.95 (1.00–3.72)
*P* value	0.449	0.007*
**LACs**		
Yes (N = 9)	2.43 (1.50–2.43)	2.68 (0.96–3.78)
No (N = 49)	1.32 (0.91–2.18)	2.68 (0.95–3.64)
*P* value	0.100	0.629
**Consumed C3** (<80 mg/dl)		
Yes (N = 28)	1.52 (1.26–2.41)	2.58 (0.98–3.78)
No (N = 30)	1.29 (0.90–2.41)	2.68 (0.95–3.64)
*P* value	0.245	0.311
**Consumed C4 (<12 mg/dl)**		
Yes (N = 22)	1.51 (1.21–2.18)	1.29 (0.95–3.78)
No (N = 36)	1.50 (0.91–2.42)	2.72 (0.95–3.64)
*P* value	0.955	0.786
**CRP positivity (>6mg/mL)**		
Yes (N = 6)	1.42 (1.21–1.62)	2.29 (0.94–3.67)
No (N = 52)	1.50 (0.91–2.41)	2.68 (0.95–3.65)
*P* value	0.718	0.609
**SLEDAI score**		
In remission (N = 15)	1.52 (0.90–2.41)	2.72 (0.95–3.64)
Mild (N = 18)	1.52 (1.23–2.41)	3.30 (2.68–3.78)
Moderate (N = 16)	1.32 (1.10–2.29)	1.29 (0.98–2.65)
High (N = 6)	0.91 (0.80–1.50)	2.35 (0.93–4.29)
Very High (N = 3)	2.43 (0.81–0.243)	0.96 (0.95–0.96)
*P* value	0.425	0.117
**SLEDAI score**		
In remission (N = 15)	1.52 (0.90–2.41)	2.72 (0.95–3.64)
Mild (N = 18)	1.52 (1.23–2.41)	3.30 (2.68–3.78)
Moderate (N = 16)	1.32 (1.10–2.29)	1.29 (0.98–2.65)
High (N = 6)	0.91 (0.80–1.50)	2.35 (0.93–4.29)
Very High (N = 3)	2.43 (0.81–0.243)	0.96 (0.95–0.96)
*P* value	0.425	0.117

Data are expressed as median (IQR), Mann-Whitney U test or Kruskal Wallis test was used.

SLE: Systemic lupus erythematosus, anti-dsDNA: anti-double stranded DNA, aCL: anticardiolipin, LACs: Lupus anticoagulants, C3: complement 3, C4: complement 4, CRP: C-reactive protein, SLEDAI: Systemic Lupus Erythematosus Disease Activity Index.

* Significant at *p* value<0.05.

### Correlation between serum expression levels of lncRNA-Cox2 and HOTAIR and clinical manifestations and laboratory findings in SLE patients

The study demonstrated a significant positive correlation between serum levels of lncRNA-Cox2 and each of IL-6 (r = 0.308, P = 0.019) and MMP-9 (r = 0.396, P = 0.002). Besides, serum level of HOTAIR had a significantly positive correlation with IL-6 (r = 0.675, P<0.0001), MMP-9 level (r = 0.757, P<0.0001), proteinuria (r = 0.287, P = 0.035) and grades of nephritis (r = 0.296, P = 0.024) (**Table 3**).

**Table 3:** Correlation between serum expression levels of lncRNA-Cox2 and HOTAIR and other laboratory characteristics in systemic lupus erythematosus (SLE) patients.

	lncRNA-Cox2 fold change	HOTAIR fold change
	r (P-value)	
**IL-6 (pg/ml)**	0.308 (0.019*)	0.675 (<0.0001*)
**MMP-9 (ng/ml)**	0.396 (0.002*)	0.757 (< 0.0001*)
**ESR**	0.246 (0.063)	-0.071 (0.597)
**SLEDAI**	0.006 (0.966)	-0.121 (0.367)
**SLICC/ACR**	-0.055 (0.683)	-0.013 (0.922)
**Nephritis grades**	0.026 (0.847)	0.296 (0.024*)
**Proteinuria (gm)**	0.088 (0.534)	0.287 (0.035*)

Spearman test was used.

SLE: Systemic lupus erythematosus, ESR: Erythrocyte sedimentation rate, SLEDAI: Systemic Lupus Erythematosus Disease Activity, SLICC/ACR: Systemic Lupus International Collaborating Clinics/American College of Rheumatology (SLICC/ACR) Damage Index,

* Significance at *p* value <0.05.

### Role of lncRNA-Cox2 and HOTAIR for prediction of SLE

According to the multivariate regression analysis, lncRNA-Cox2 and HOTAIR were found as independent SLE predictors. The odds ratio, 95% confidence interval (OR, 95% CI) for lncRNA-Cox2 was 8.60 (1.66–44.48), P = 0.01. Regarding HOTAIR, the OR, 95% CI was 12.68 (2.27–70.94), P = 0.004 (**Table 4**).

**Table 4:** Multiple logistic regression analysis of systemic lupus erythematosus (SLE).

	B	SE	P-value	Odds ratio (OR)	95% CI.	
					Lower	Upper
**lncRNA-Cox2**	2.251	0.838	0.010*	8.60	1.66	44.48
**lncRNA-HOTAIR**	2.540	0.878	0.004*	12.68	2.27	70.94
**Constant**	-5.881	1.263	<0.0001	0.003		

SE, standard error of B; CI., confidence interval.

* Significance at *p* value <0.05.

**Fig 2:**
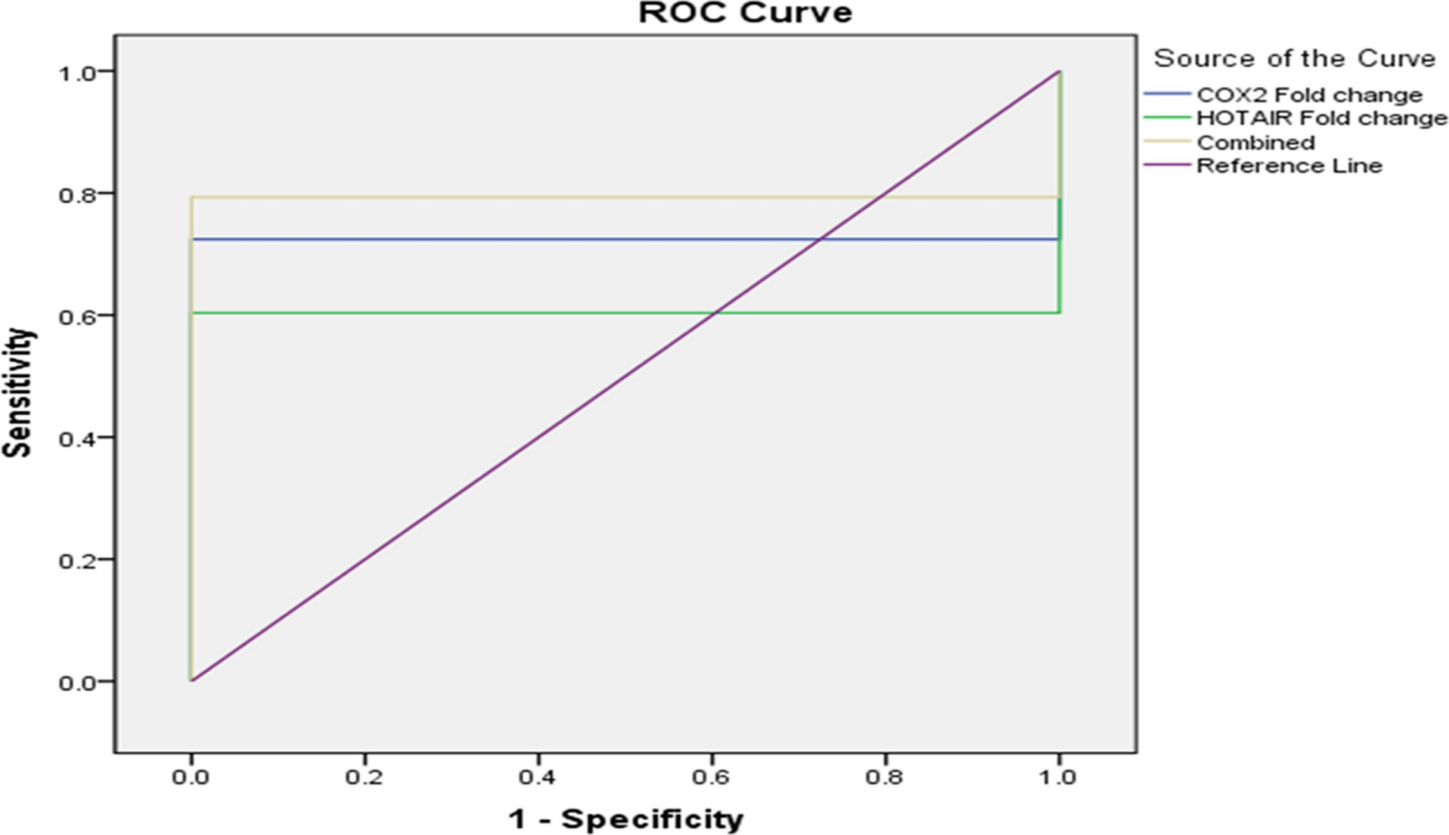
ROC curve for validation of lnc-Cox2 and HOTAIR to differentiate SLE patients from healthy subjects.

Moreover, the ROC curve was done to detect the best cut-off values with the highest sensitivities and specificities to discriminate between SLE and HCs. The AUC for lncRNA-Cox2 and HOTAIR in predicting SLE versus HCs was 0.724 and 0.603 respectively. LncRNA-Cox2 showed sensitivity and specificity 72.4%, and 100.0% respectively at the cut-off point of 1.11. HOTAIR sensitivity was 60.3%, specificity was 100.0% at the cut-off point of 1.28. It was found that the combined utilization of lncRNA-Cox2 and HOTAIR in the diagnosis of SLE to HCs had sensitivity and specificity of 79.3% and 100.0%, respectively with an AUC of 0.793 at the cut-off point of 0.26 (**Fig 2**).

## Discussion

While hard work has been done to explain the etiology of SLE, its accurate pathogenesis is still unknown. Thus, a study of the genetic and molecular aberrations of SLE is considered an important topic and is likely to be the key to identify novel biomarkers [22]. Recently, studies have also suggested that abnormal expression of lncRNAs might be associated with numerous diseases, indicating that these RNAs may present a new avenue for diagnostic and therapeutic targets by recognition of their roles in human diseases [7].

In this study, we considered the serum expression levels of lncRNA-Cox2 and HOTAIR and examined the relationship between their levels and variable clinical characters in SLE patients. Up to our knowledge, this is the first time to measure lncRNA-Cox2 in SLE patients. A significant increase in the serum expression level of lncRNA-Cox2 in SLE patients was detected when compared to HCs.

Numerous recent reports have elucidated that lncRNA-Cox2 which originates in proximity to the COX-2 gene could mediate inflammatory signaling and immune-regulatory activities [23]. Tong *et al*. demonstrated that lncRNA-Cox2 affects inflammatory gene transcription in intestinal epithelial cells [8]. Also, Carpenter *et al*. observed that lncRNA-Cox2 acts as a key regulator of immune genes expression which mediates inflammatory reaction [4]. Furthermore, it was demonstrated that TLRs could induce lncRNA-Cox2 to stimulate a variety of genes that modulate immune response [24]. In addition, it was reported that lncRNA-Cox2 helped nuclear translocation of NF-κB p65 so activating inflammatory genes transcription [25].

Notably, it was shown that lncRNA-Cox2 could act as a novel biomarker and serve as a key biomarker in rheumatoid arthritis diagnosis [26].

The lncRNA-Cox2 expression level was associated with the presence of neurological manifestations in SLE patients. Although no previous reports had linked LncRNA-Cox2 to neurological manifestations related to SLE, its increased expression level has been well-known in many neurological disorders. It was reported that lncRNA-Cox2 participates in the pathogenesis of Alzheimer’s disease through its interactions with heterogeneous nuclear; ribonucleoprotein A/B and A2/B1 essential for target gene inhibition [27]. Additionally, we detected a significant positive correlation between lncRNA-Cox2 and IL-6 as well as MMP-9 in patients with SLE. These results may explain the crucial role of lncRNA-Cox2 in regulating inflammatory-mediated pathways in SLE.

In this study, a positive significant correlation between serum levels of lncRNA-Cox2 and IL-6 was in line with the previous study that found that lncRNA-Cox2 is important for induction of some genes including the IL-6 gene following the Pam3CSK4 treatment [4] and another study reported that knockdown of lncRNA-Cox2 severely impaired the production of the proinflammatory cytokine IL-6 [28]. However, the precise molecular mechanism by which lncRNACox2 mediates these effects is still unclear. Also, different studies have reported that the inflammatory cytokines such as IL-6 and TNF-α had a key role in SLE pathogenesis and these markers may be increased due to other pathogenic mechanisms [14, 29, 30].

In the current study, HOTAIR was significantly up-regulated in SLE patients in comparison with HCs. Wu and colleagues have reported that the high plasma expression levels of HOTAIRM1 (HOX antisense intergenic RNA myeloid 1) in patients with SLE were not significantly different from that of the HCs [31]. HOTAIRM1 is a myeloid-specific that plays an important role in the maturation of granulocytes [32]. A recent study has shown that PU1 (a transcription factor) which has been involved in the pathogenesis of SLE, controls the HOTAIRM1 expression during differentiation of granulocytes [33]. It is still not well understood if the dysregulation of HOTAIR could have a role in SLE pathogenesis. B cells, T cells and dendritic cells, which are sources of HOTAIR, are essential cells for the pathogenesis of SLE [34]. However, the molecular and cellular pathogenic mechanisms that involve lncRNA are still undefined [35].

In this study, a positive significant correlation was demonstrated between serum levels of HOTAIR and each of IL-6 and MMP-9 levels. This agrees with the work of Wang *et al*. who found that down-regulated HOTAIR leads to suppression of MMP2 and MMP9 secretion. They also concluded that in-vitro and in-vivo knockdown of HOTAIR significantly decreased MMP2 and MMP9 levels [15]. Thus, we can associate the increased expression of HOTAIR with the increased levels of MMP-9 in our SLE patients. MMP-9 was considered as an inflammatory marker detected at elevated levels in the sera of SLE patients in comparison to HCs in many earlier studies [36, 37].

The significantly increased levels of MMP-9 may also be explained by the role of the activated transcriptional factor nuclear factor-κB in the immune response, together with the role of T-cells in SLE, which increase the transcription activity of many cytokines participating in induction of inflammation and stimulation of MMPs proteins expression [38].

In the current study, the lncRNA-Cox2 and HOTAIR were highly sensitive and specific in differentiating patients with SLE from HCs using the ROC curve. As novel biomarkers, lncRNAs have the following characteristics. First, lncRNAs are highly stable in the samples compared to mRNAs of protein-coding genes [39]. Second, they are highly tissue-specific compared with protein-coding mRNAs, which are expressed in many tissues and differentially expressed in variable diseases [10]. In addition, lncRNAs are also detectable in body fluids such as serum and urine [40, 41] which are easy to collect using non-invasive methods. These features make lncRNAs very suitable as non-invasive biomarkers. Unlike micro RNAs, lncRNAs show greater complexity of their functions and have wider biological activities [42].

## Conclusion

We have concluded that lncRNA-Cox2 and HOTAIR may serve as potential non-invasive biomarkers for the diagnosis of patients with SLE. Accordingly, they could be considered as SLE treatment targets. Exploring the role of lncRNAs in SLE pathogenesis is essential for the deep understanding and consequently effective treatment of this disease. Limitations in our study include: (i) The participation and/or dysregulation of lncRNAs in SLE pathogenesis are needed to be investigated in specific organs and cell types, (ii) The population of enrolled patients was relatively small, so we need a larger sample size to verify our results, (ii) further studies are needed to be conducted on different ethnic groups, (iii) More investigations are required to define the exact molecular mechanisms by which lncRNA-Cox2 and HOTAIR are involved in SLE pathogenesis.

## Supporting information

S1 Table Fold change levels of serum lncRNA-Cox2 and lncRNA-HOTAIR in systemic lupus erythematosus patients. Data are expressed as Median (IQR), Mann-Whitney U test was used. Expression levels in the healthy group are equivalent to 1. * Significant at *p* value<0.05.
(DOCX)

S2 Table Validity of lnc-Cox2 and HOTAIR for differentiation between systemic lupus erythematosus patients and healthy subjects. (DOCX)
