# Supplementary material for: The potential role of serum expression profile of long non coding RNAs, Cox2 and HOTAIR as novel diagnostic biomarkers in systemic lupus erythematosus
Source: PLoS One. 2022 Aug 16;17(8):e0268176. doi: 10.1371/journal.pone.0268176 (PMC9380942; doi:10.1371/journal.pone.0268176)
Supplement: S1 Table — Data are expressed as Median (IQR), Mann-Whitney U test was used. Expression levels in the healthy group are equivalent to 1. * Significant at p value<0.05. (DOCX) [file pone.0268176.s001.docx]

**Table S1.** Fold change levels of serum lncRNA-Cox2 and lncRNA-HOTAIR in systemic lupus erythematosus patients.

|  | **Median (IQR)** | **P value** |
| --- | --- | --- |
| **COX Fold change** | 1.29 (0.81-1.71) | **<0.0001*** |
| **HOTAIR Fold change** | 2.68 (0.95-3.67) | **0.038*** |

Data are expressed as Median (IQR), Mann-Whitney U test was used. Expression levels in the healthy group are equivalent to 1.

* Significant at *p* value<0.05.
