# Supplementary material for: The potential role of serum expression profile of long non coding RNAs, Cox2 and HOTAIR as novel diagnostic biomarkers in systemic lupus erythematosus
Source: PLoS One. 2022 Aug 16;17(8):e0268176. doi: 10.1371/journal.pone.0268176 (PMC9380942; doi:10.1371/journal.pone.0268176)
Supplement: S2 Table — (DOCX) [file pone.0268176.s002.docx]

**Table S2**. Validity of lnc-Cox2 and HOTAIR for differentiation between systemic lupus erythematosus patients and healthy subjects.

|  | **AUC** | **Cut-off point** | **Sensitivity** | **Specificity** |
| --- | --- | --- | --- | --- |
| **lncRNA-Cox2** | **0.724** | 1.11 | 72.4 | 100.0 |
| **lncRNA-HOTAIR** | 0.603 | 1.28 | 60.3 | 100.0 |
| **Combined** | **0.793** | 0.26 | 79.3 | 100.0 |
